# Fast Maturation of Splenic Dendritic Cells Upon TBI Is Associated With FLT3/FLT3L Signaling

**DOI:** 10.3389/fimmu.2022.824459

**Published:** 2022-02-24

**Authors:** Jin Zhang, Zhenghui Li, Akila Chandrasekar, Shun Li, Albert Ludolph, Tobias Maria Boeckers, Markus Huber-Lang, Francesco Roselli, Florian olde Heuvel

**Affiliations:** ^1^ Department of Neurology, Center for Biomedical Research (ZBMF), Ulm University, Ulm, Germany; ^2^ Department of Neurosurgery, Kaifeng Central Hospital, Kaifeng, China; ^3^ German Center for Neurodegenerative Diseases (DZNE) , Ulm, Germany; ^4^ Institute of Anatomy and Cell Biology, Ulm University, Ulm, Germany; ^5^ Institute of Clinical and Experimental Trauma-Immunology, University Hospital, Ulm, Germany

**Keywords:** traumatic brain injury, spleen, FLT3, dendritic cell, ethanol

## Abstract

The consequences of systemic inflammation are a significant burden after traumatic brain injury (TBI), with almost all organs affected. This response consists of inflammation and concurrent immunosuppression after injury. One of the main immune regulatory organs, the spleen, is highly interactive with the brain. Along this brain–spleen axis, both nerve fibers as well as brain-derived circulating mediators have been shown to interact directly with splenic immune cells. One of the most significant comorbidities in TBI is acute ethanol intoxication (EI), with almost 40% of patients showing a positive blood alcohol level (BAL) upon injury. EI by itself has been shown to reduce proinflammatory mediators dose-dependently and enhance anti-inflammatory mediators in the spleen. However, how the splenic immune modulatory effect reacts to EI in TBI remains unclear. Therefore, we investigated early splenic immune responses after TBI with and without EI, using gene expression screening of cytokines and chemokines and fluorescence staining of thin spleen sections to investigate cellular mechanisms in immune cells. We found a strong *FLT3*/*FLT3L* induction 3 h after TBI, which was enhanced by EI. The *FLT3L* induction resulted in phosphorylation of FLT3 in CD11c+ dendritic cells, which enhanced protein synthesis, maturation process, and the immunity of dendritic cells, shown by pS6, peIF2A, MHC-II, LAMP1, and CD68 by immunostaining and TNF-α expression by *in-situ* hybridization. In conclusion, these data indicate that TBI induces a fast maturation and immunity of dendritic cells which is associated with FLT3/FLT3L signaling and which is enhanced by EI prior to TBI.

## Introduction

Traumatic injury to the brain has acute, large-scale systemic consequences (1) that affect almost all organs and may lead to a compromised function of the heart, lung, gastrointestinal tract, liver, kidney, bones, lymphoid organs, and others, without direct systemic injury or infection (2, 3). The systemic response to TBI is characterized by inflammation and, at the same time, a net systemic immunosuppression (4–6). Although brain injury results in a systemic increase of inflammatory mediators and cytokines in both patients (7–9) and rodent models of TBI (10–12), there remains ample evidence pointing toward systemic immunosuppression post-TBI, with a decrease in immune cells in the periphery (13–18). Generalized immunosuppression is highly relevant on clinical grounds, since it may contribute to an enhanced vulnerability to infections observed after severe tissue injury.

The spleen is one of the most important immune regulatory organs, involved not only in red blood cell clearance but also in the facilitation of interactions between antigen-presenting cells (APCs) and T and B lymphocytes (19). Prior investigation of the brain–spleen axis has revealed the interaction and regulation of splenic responses initiated by the central nervous system by either circulating mediators whose receptors are located on APCs (20) or by autonomic nerve fibers associated with splenic immune cells (21). There have been reports of sympathetic and parasympathetic fibers innervating dendritic cells (DCs) in the spleen (22). Furthermore, vagus nerve stimulation can reduce macrophage-induced TNF alpha release in the spleen through a so-called cholinergic anti-inflammatory pathway (23). However, it remains poorly understood whether the spleen is directly and rapidly involved in the early response to TBI.

Ethanol intoxication (EI) is a frequent comorbidity in brain injury, with almost 40% of patients showing a positive blood alcohol level (BAL) upon admission (24). Notably, the largest majority of patients showing EI in the context of TBI are not chronic alcohol consumers but rather young and often episodic weekend drinkers (the so-called “drink-and-drive” patients). Recent studies revealed that acute EI can have beneficial effects on the neuroimmunological response following experimental TBI (25–27), these findings have been supported by clinical evidence (28–30). However, some clinical studies have reported the opposite (31, 32). Animal models have demonstrated that high doses of EI reduce spleen size (33) and pro-inflammatory cytokines IL-1β and IL-6 but increase the anti-inflammatory cytokine IL-10 (34). Furthermore, EI has been shown to suppress antigen presentation by DCs (35), reflecting the immunosuppressive effects of ethanol intoxication on the spleen.

Is acute EI posited to interfere or rather to amplify the TBI-induced systemic immunoregulation, and in particular, is EI modulating TBI-induced immune reactions in the spleen? Given the high prevalence of EI in TBI patients, this question has direct clinical relevance. In this study, we investigated the effect of a single high-dose ethanol exposure prior to experimental TBI in adult mice with a focus on the immediate immunological responses in the spleen.

## Material and Methods

### Animals, Traumatic Brain Injury Model, and Ethanol Treatment

This study represents a *post-hoc* analysis of spleen samples obtained from previous studies (25, 26, 36, 37). Investigations with these samples have never been reported before, and this study was undertaken in accordance with the 3R principle, to reduce the number of mice in animal experimentation but increase the scientific output from animal sacrifice. These experiments have been approved by Ulm University Animal Experimentation Oversight Committee and by the Regierungspräsidium Tübingen (license number 1222). Male wild-type mice (B6-SJL) were bred locally under standard housing conditions (24°C, 60%–80% humidity, 12/12 light/dark cycle, with *ad libitum* access to food and water). TBI was performed on wild-type (WT) male mice aged p60–90, in agreement with epidemiological data in human TBI (38–40). Experimental TBI was performed as previously reported (25, 26, 36, 37). Briefly, mice were administered buprenorphine (0.1 mg/kg by subcutaneous injection) and anesthetized with sevoflurane (2.5% in 97.5% O_2_), after which the mice were subjected to a closed head weight drop TBI model. Animals were positioned in the weight-drop apparatus, and the TBI was delivered by a weight of 333 g free falling from a height of 2 cm, targeting the parietal bone (41). Directly after the TBI, mice were administered 100% O_2,_ and apnea time was monitored. Control mice (sham group) had the same treatment and procedures (analgesia, anesthesia, skin incision, and handling), but without the trauma being administered. Ethanol treatment was performed as previously described (42, 43). Briefly, 100% synthesis grade ethanol was diluted in 0.9% NaCl saline to a final dilution of 32% volume/volume (32 µl of 100% ethanol and 68 µl of saline). Mice (20–25 g) were administered a volume of 400–500 µl of diluted ethanol (to obtain a concentration of 5 g/kg) by oral gavage 30 min before TBI. Four experimental groups were investigated: saline administered, subjected to sham surgery (saline-sham, SS); saline administered, subjected to TBI (saline-TBI, ST); ethanol administered, subjected to sham surgery (ethanol-sham, ES); and ethanol administered, subjected to TBI (ethanol-TBI, ET).

### Tissue Isolation

Three hours post-trauma, mice were euthanized by cervical dislocation and organs were harvested for further processing. The spleen was dissected quickly and snap frozen in dry ice for further processing. Tissue was used for either RNA isolation and quantitative RT-PCR, for tissue sectioning and immunofluorescence staining.

### RNA Isolation and Quantitative RT-PCR

RNA was isolated from the spleen using QIAzol (Qiagen, Germany) by disrupting and homogenizing the tissue in 1 ml QIAzol, after which 200 µl of chloroform was added and vortexed for 15 s. The samples were placed at RT for 10 min and centrifuged for 10 min 12,000×*g* at 4°C to achieve phase separation. The top layer (containing RNA) was moved to another tube and precipitated with the same amount of isopropanol. The samples were placed at RT for 10 min and centrifuged for 10 min 12,000×*g* at 4°C. The isopropanol was removed and 1 ml of 75% ethanol in DEPC-treated dH_2_O was added and mixed. The samples were centrifuged for 10 min 8,000×*g* at 4°C, ethanol was removed, and the samples were air dried. The RNA pellet was redissolved in 20 µl RNAse-free dH_2_O. RNA concentration was determined by NanoDrop. Reverse transcription was performed by adding 5 µl random hexamers (Biomers, Germany) to 0.75μg RNA (total volume 40 µl diluted in dH_2_O) and incubated for 10 min at 70°C. The samples were placed on ice and a master mix of 0.5µl reverse transcriptase (Promega, Germany), 0.5 µl RNase Inhibitor (RiboLock, Thermo Scientific, Germany), 2 µl dNTPs (Genaxxon, Germany), and 12 µl reverse transcriptase buffer (Promega, Germany). The samples were incubated for 10 min at RT and placed in a water bath for 45 min at 42°C. The samples were incubated for 3 min at 99°C, placed on ice, and frozen until further use.

The primers used in the present study were designed using the primer blast tool from NCBI (National Center for Biotechnology Information, USA), and sequences were found through the NCBI nucleotide search tool, GenBank. Sequences were copied into the primer blast tool and parameters were set to achieve the most optimal PCR product for the in-house lightcycler (Roche LightCycler 480 II). Briefly, the PCR product size was set from a minimum of 70 to a maximum of 140, the primer melting temperature (Tm) was set at 60°C ± 3°C, the exon junction span was set at no preference, and finally, the correct organism (*Mus musculus*) was set. The primer pair with the most optimal Tm, self-complementarity, and self-3′ complementarity was chosen. The chosen primer pairs were double-checked in the primer blast tool, to ensure specificity for the target gene. Primers were ordered from Biomers (Germany) and were validated, by performing a run on test samples together with the corresponding controls (samples without RNA and samples without reverse transcriptase) to verify the Ct value and thereby the selectivity and functioning of the primer before using them for the experiments. The detailed list of the primer sequences for each gene tested is reported in **Supplementary Table 1**.

qPCR was performed on the LightCycler 480 II (Roche) with the Power PCR TB green PCR master mix (Takara, Japan). Two microliters of sample cDNA was used in a total volume of 10 µl (3 µl primer mix and 5 µl of TB green) in a 96-well plate, all samples were duplicated, and the housekeeping gene GAPDH was used as a control (for a complete overview of cytokine sequences, see **Supplementary Table 1**). The Ct values obtained from the lightcycler were normalized according to the following equation: 2−ΔCt (ΔCt = Cttarget gene − CtGapdh) = relative mRNA.

### Tissue Sectioning and Immunofluorescence Staining

Frozen spleen tissue was embedded in OCT (Tissue-Tek, The Netherlands), and 10 μm sections were cut with the cryostat and mounted on glass slides. Slides were stored for 24 h at −80°C and washed in 1× PBS, followed by a 10-min fixation step of the sections in 4% PFA. Target retrieval was performed in sodium citrate buffer pH 8.5, followed by blocking of the sections in blocking buffer (3% BSA, 0.3% Triton X-100; PBS) for 2 h at RT. The primary antibodies (for a complete overview of antibodies used, see **Supplementary Table 2**) were diluted in blocking buffer and incubated for 48 h at 4°C, followed by 3 × 30 min washes in PBS at RT. The secondary antibodies were diluted in blocking buffer and incubated for 2 h at RT, followed by 3 × 30 min washes in PBS, and the sections were mounted using Fluorogold prolong antifade mounting medium (Invitrogen, Germany).

### Single mRNA *In-Situ* Hybridization

Fluorescence *in-situ* mRNA hybridization was used as previously reported (44) in agreement with the manufacturer’s instructions (ACDBio, RNAscope, Newark, CA, USA fluorescence *in-situ* hybridization for fresh frozen tissue, all reagents and buffers were provided by ACDBio), with small modifications (25). Briefly, frozen spleen tissue was embedded in OCT (Tissue-Tek, the Netherlands), and 10 μm sections were cut with the cryostat and mounted on Superfrost Plus glass slides. Slides were stored for 24 h at −80°C and fixed for 10 min in 4% PFA at 4°C. Sections were covered by protease IV and incubated for 30 min at 40°C followed by 2 × 2 min washing in PBS. Both probes (TNF-α and ADRB2) were added and incubated for 4.5 h at 40°C followed by 2 × 2 min washing step with wash buffer. Then, amplification 1 was added to the sections and incubated for 30 min at 40°C followed by 2 × 2 min washing step with wash buffer. Next, amplification 2 was added to the sections and incubated for 15 min at 40°C followed by 2 × 2 min washing step with wash buffer. As a final amplification step, amplification 3 was added and incubated for 30 min at 40°C followed by 2 × 2 min washing step with wash buffer. Finally, the detection step was performed by adding detection reagent 4A to the sections and incubated for 45 min at 40°C followed by 2 × 2 min washing step with wash buffer, and then, the sections were blocked in blocking buffer (3% BSA, 0.3% Triton X-100; PBS) for 1 h at RT followed by an overnight incubation with primary antibodies diluted in blocking buffer. The sections were washed 3 × 30 min in PBST and incubated with secondary antibodies diluted in blocking buffer for 2 h at RT. A final washing step 3 × 30 min in PBST was performed and the sections were counterstained with DAPI and mounted using Fluorogold prolong antifade mounting medium (Invitrogen, Germany).

### Image Acquisition and Image Analysis

Immunofluorescence staining was imaged with a Keyence BZ-X800 microscope (Keyence, Japan) equipped with a ×100 oil objective, and a single optical section with 3 × 3 tile scan was made spanning an area covering a splenic follicle and the red pulp. Acquisition parameters were set to avoid hyper- or hyposaturation and kept constant for each experimental set. Images were merged with the BZ-II analyzer software (Keyence, Japan) and analyzed using the ImageJ software. Fluorescence intensity was assessed by manually tracing the CD11c+ cells and measuring the mean gray value. Density was assessed by using the ImageJ plugin cell counter. The analyzed marker was assessed as high or low expressing, by thresholding the signal. Each picture was analyzed by making a ratio between total CD11c+/CD45+ cells and the imaged marker or the ratio between CD11c−/CD45+ cells and the imaged marker.

Single mRNA *in-situ* hybridization images were acquired using an LSM-710 (Carl Zeiss, Germany) microscope with a ×40 oil objective with optical thickness fitted to the optimum value. A z-stack of 8 images were acquired at 1,024 × 1,024 pixel resolution and 16-bit depth. Acquisition parameters were set to avoid over- and undersaturation and kept the same for each experimental set. TNF-α and beta-2 adrenergic receptor (ADRB2) mRNA density in CD11c+ cells were assessed by using the ImageJ plugin cell counter.

### Statistical Analysis

**Figure 1:**
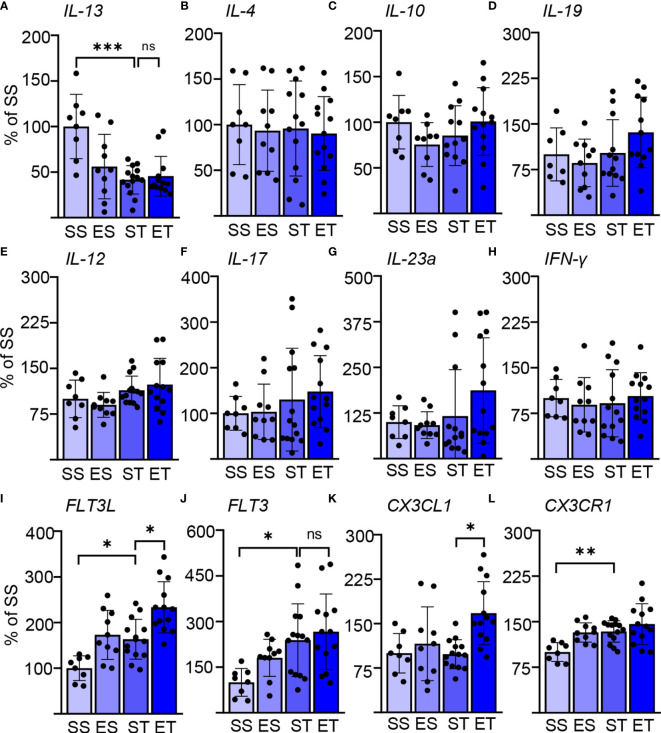
Ethanol intoxication (EI) enhances the selective cytokine expression after traumatic brain injury (TBI). Cytokine expression screening in the spleen of saline sham (SS), ethanol sham (ES), saline TBI (ST), and ethanol TBI (ET)-treated mice 3 h after trauma. **(A–D)** Bar plots show the relative expression of Th2 cell and anti-inflammatory markers: *IL-13*, *IL-4*, *IL-10*, and *IL-19*. *IL-13* expression showed a significant downregulation after TBI (SS vs. ST; *p* < 0.0005); ethanol pretreatment did not alter the TBI-induced effect on *IL-13* (ST vs. ET; *p* > 0.05). *IL-4*, *IL-10*, and *IL-19* were not affected by any treatment. **(E–H)** Bar plots show the relative expression of Th1 cell and pro-inflammatory markers: *IL-12*, *IL-17*, *IL-23a*, and *IFN-y*. TBI with or without ethanol pretreatment showed no significant differences. **(I–L)** Bar plots show the relative expression of dendritic cell (DC)–monocyte-specific mediators: *FLT3*, *FLT3R*, *CX3CL1*, and *CX3CR1*. TBI resulted in a significant increase of *FLT3* (SS vs. ST; *p* < 0.037), *FLT3L* (SS vs. ST; *p* < 0.045), and *CX3CR1* (SS vs. ST; *p* < 0.01). Ethanol pretreatment resulted in a further significant enhancement of *FLT3* (ST vs. ET; *p* < 0.026) and *CX3CL1* (ST vs. ET; *p* < 0.006). Data shown as bar plots and individual data points. Group size: SS *N* = 8, ES *N* = 10, ST *N* = 14, ET *N* = 13. *p < 0.05; **p < 0.01; ***p < 0.001; ns, not significant.

**Figure 2:**
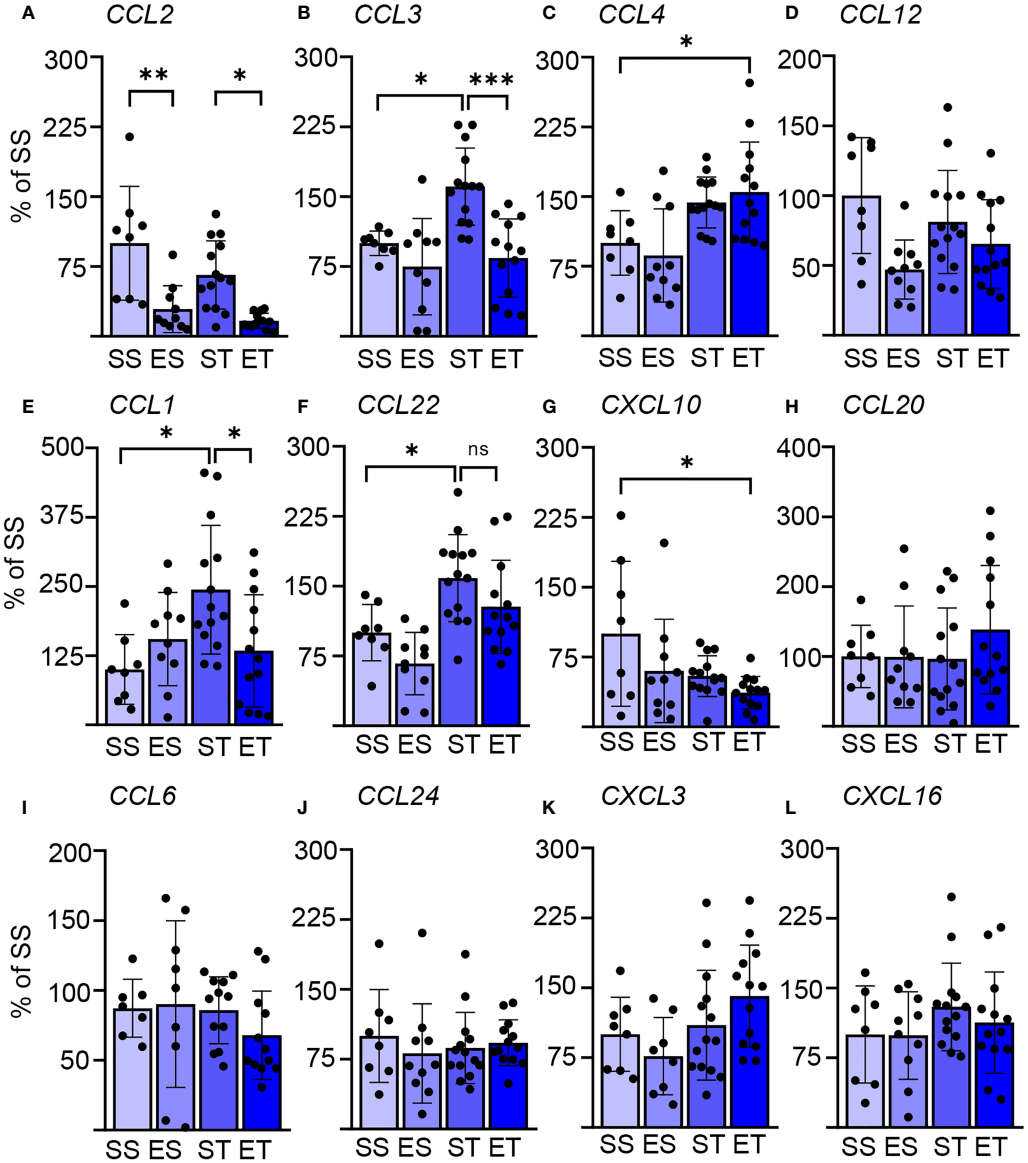
Splenic chemokine expression shows a fast response to TBI and EI. Chemokine expression data in the spleen of saline sham (SS), ethanol sham (ES), saline TBI (ST), and ethanol TBI (ET)-treated mice 3 h after trauma. **(A–D)** Bar plots show the relative expression of modulators of DC biology: *CCL2*, *CCL3*, *CCL4*, and *CCL12*. TBI resulted in a significant upregulation of *CCL3* (SS vs. ST; *p* = 0.028), and ethanol pretreatment significantly reduced the TBI-induced *CCL3* expression (ST vs. ET; *p* = 0.0006). Ethanol by itself resulted in a significant downregulation of *CCL2*, independent of TBI (SS vs. ES, *p* = 0.0021; ST vs. ET, *p* = 0.014). ET resulted in a significant upregulation of *CCL4* compared with SS (SS vs. ET; *p* = 0.042). *CCL12* expression was not affected by any treatment. **(E–H)** Bar plots show the relative expression of modulators of Th cells: *CCL1*, *CCL22*, *CXCL10*, and *CCL20*. TBI resulted in a significant upregulation of *CCL1* (SS vs. ST; *p* = 0.019) and *CCL22* (SS vs. ST; *p* = 0.039), and ethanol pretreatment significantly reduced the TBI-induced *CCL1* expression (ST vs. ET; *p* = 0.037) but not for *CCL22* (ST vs. ET; *p* = 0.35). ET resulted in a significant downregulation of *CXCL10* compared with SS (SS vs. ET; *p* = 0.014). *CCL10* expression was not affected by any treatment. **(I–L)** Bar plots show the relative expression of modulators of leukocytes and NK cells: *CCL6*, *CCL24*, *CXCL3*, and *CXCL16*. TBI with or without ethanol pretreatment showed no significant differences. Data shown as bar plots and individual data points. Group size: SS *N* = 8, ES *N* = 10, ST *N* = 14, ET *N* = 13. *p < 0.05; **p < 0.01; ***p < 0.001.

Statistical analysis for the gene expression data sets (**Figures 1**, **2**) was performed using the IBM software suite, using a two-way multivariate ANOVA (with Wilk’s *λ* parameter), because the experiment included multiple dependent variables (cytokines or chemokines) and two independent variables (TBI and ethanol treatment). The *post-hoc* comparisons were performed with two-way ANOVA with Tukey’s *post-hoc* correction. All groups were tested for normality using the Shapiro–Wilk test. Correlation matrices of gene expression data were made with the Prism analysis suite (GraphPad Prism version 8), and Pearson *r* coefficient and *p*-values for every correlation were assessed. For the histological datasets, two-way ANOVA was performed with Tukey’s *post-hoc* comparison, since two independent treatments were done (saline/ethanol and sham/TBI). Statistical significance was set at *p <*0.05 after multiple-comparison correction.

## Results

### TBI Induces a Rapid and Selective Induction of Cytokine Upregulation in the Spleen, Enhanced by Concomitant EI

Systemic immune functions are quickly modulated by the occurrence of neurological conditions through pathways conceptualized as the “brain–spleen axis” (45–47). We set out to verify if any rapid modification in the splenic immune responses would take place upon mild/moderate TBI and, most importantly, if the comorbidity of EI would significantly interact with such responses.

As an entry point, we screened the induction of the mRNA of several cytokines (with the acknowledged limitation that mRNA levels may not directly represent protein levels) in whole-spleen extracts obtained 3 h after TBI (or sham surgery), pretreated (30 min) either with saline or with ethanol (5 g/kg).

We considered three sets of cytokines: prototypical Th1-cellular-immunity-directed, pro-inflammatory mediators (*IL-12*, *IL-17*, *IL-23a*, and *IFN-y*); prototypical Th2-B-cell-directed, anti-inflammatory mediators (*IL-4*, *IL-10*, *IL-13*, and *IL-19*); and prototypical DC–monocyte mediators (*FLT3*, *FLT3L*, *CX3CL1*, and *CX3CR1*) (48–51). Surprisingly, we identified a significant effect of both the TBI itself and EI (two-way MANOVA; TBI: *F* = 7.379, Wilks’ *λ* = 0.253, *p* = 0.0001; EI: *F* = 4.599, Wilks’ *λ* = 0.352, *p* = 0.0001), and the interaction of the two parameters showed no significance in the two-way MANOVA (*F* = 1.801, Wilks’ *λ* = 0.581, *p* = 0.09). This could be attributed (two-way ANOVA, Tukey corrected) to the significantly increased expression of *FLT3L* upon TBI alone (*F*_(3, 34)_ = 9.771; SS vs. ST; 100 ± 27 vs. 163 ± 44; *p* = 0.045; **Figure 1I**), *FLT3* (*F*_(3, 36)_ = 4.266; SS vs. ST; 100 ± 46 vs. 239 ± 119; *p* = 0.037; **Figure 1J**), and *CX3CR1* (*F*_(3, 41)_ = 6.825; SS vs. ST; 100 ± 16 vs. 134 ± 18; *p* = 0.01; **Figure 1L**), whereas *IL-13* was significantly downregulated (*F*_(3, 37)_ = 7.499; SS vs. ST; 100 ± 35 vs. 42 ± 16; *p* = 0.0005; **Figure 1A**). Ethanol intoxication before TBI resulted in a significant further enhancement of the expression of *FLT3L* (ST vs. ET; 163 ± 44 vs. 233 ± 56; *p* = 0.026; **Figure 1I**) and *CX3CL1* (ST vs. ET; 98 ± 25 vs. 168 ± 53; *p* = 0.006; **Figure 1K**), whereas *FLT3* and *CX3CR1* were unaltered in ET compared with ST (*FLT3*: ST vs. ET, 239 ± 119 vs. 266 ± 124, *p* = 0.93, **Figure 1J**; *CX3CR1*: ST vs. ET, 134 ± 18 vs. 146 ± 34, *p* = 0.52, **Figure 1L**). Most notably, no effect was observed on any of the other cytokines considered.

These screening data not only point toward a rapid activation of splenic immune cells upon TBI but also reveal a pattern compatible with a selective effect on innate immunity.

### Rapid Modulation of the Splenic Chemokine Pattern by TBI and by EI/TBI

We sought to confirm and further extend the rapid effect of TBI on splenic immune cells, particularly on DCs. We assessed the expression of a set of chemokines known to be strong modulators of innate immune responses (52, 53). The focus was set, particularly on DC biology (and other APCs: *CCl2*, *CCL3*, *CCl4*, and *CCL12*) (54–56), Th cells (*CCL1*, *CCL20*, *CCL22*, and *CXCL10*) (57–60), or leukocytes and NK cells (*CCL6*, *CCl24*, *CXCL3*, and *CXCL16*) (61–64). We could identify a significant effect of TBI (two-way MANOVA: *F* = 4.494, Wilks’ *λ* = 0.357, *p* = 0.0001) and EI (*F* = 4.956, Wilks’ *λ* = 0.335, *p* = 0.0001), and the interaction of the two parameters showed no significant effect in the two-way MANOVA (*F* = 1.776, Wilks’ *λ* = 0.585, *p* = 0.099). However, *post-hoc* analysis (Tukey corrected) showed that TBI alone significantly increased the expression of *CCL3* (*F*_(3, 37)_ = 8.910; SS vs. ST; 100 ± 13 vs. 161 ± 42; *p* = 0.028; **Figure 2B**), *CCL1* (*F*_(3, 39)_ = 4.156; SS vs. ST; 100 ± 63 vs. 244 ± 116; *p* = 0.019; **Figure 2E**), and *CCL22* (*F*_(3, 37)_ = 7.483; SS vs. ST; 100 ± 30 vs. 159 ± 47; *p* = 0.039; **Figure 2F**). On the other hand, concomitant EI significantly downregulated *CCL3* (ST vs. ET; 161 ± 42 vs. 84 ± 42; *p* = 0.0006; **Figure 2B**) and *CCL1* (ST vs. ET; 244 ± 116 vs. 134 ± 101; *p* = 0.037; **Figure 2E**), but *CCL22* was unaffected by EI and remained upregulated (ST vs. ET; 159 ± 47 vs. 128 ± 50; *p* = 0.35; **Figure 2F**). The ET group presented a distinct chemokine profile: a significant upregulation of *CCL4* was observed only in the ET group but not in the ST and ES groups alone (*F*_(3, 39)_ = 6.093; SS vs. ET, 100 ± 35 vs. 155 ± 54, *p* = 0.042; **Figure 2C**). Conversely, the ET group displayed the downregulation of *CXCL10* expression (*F*_(3, 41)_ = 3.458; SS vs. ET; 100 ± 78 vs. 37 ± 18; *p* = 0.014; **Figure 2G**). Finally, ethanol exposure alone caused a downregulation of *CCL2* in both sham and TBI groups (*F*_(3, 36)_ = 9.679; SS vs. ES, 100 ± 61 vs. 29 ± 25, *p* = 0.0021; ST vs. ET, 66 ± 37 vs. 17 ± 8, *p* = 0.014; **Figure 2A**).

As an additional exploration of the complex immunological interactions taking place in the spleen upon TBI with or without concomitant EI, we performed a cross-correlation analysis of the expression levels of the tested cytokines and chemokines (**Supplementary Data File 1**). We detected a significant correlation among the expression levels of *IL-17*, *IL-23*, *IL-19*, and *IFN-γ* (known to constitute a unified pathway) (65, 66) in ST and ET samples, underscoring the relevance of this cytokine module in TBI. Interestingly, *FLT3* and *FLT3L* expression was correlated in ET but not in ST samples. Furthermore, the expression levels of several chemokines (such as *CCL2*, *CCL3*, and *CCL4* vs. *CCL1*, *CCL20*, and *CCL24*) were significantly correlated in ET but not in ST samples, further underscoring the peculiar immunological milieu determined by the concomitant TBI and EI.

These data not only confirm the rapid engagement of the brain–spleen axis upon TBI but also show that EI displays a substantial modulatory effect on innate and adaptive immunity, particularly on APCs.

### TBI Induces the Phosphorylation of FLT3 and Its Downstream Signaling Partner BTK in DCs, Which Are Both Enhanced by EI

**Figure 3:**
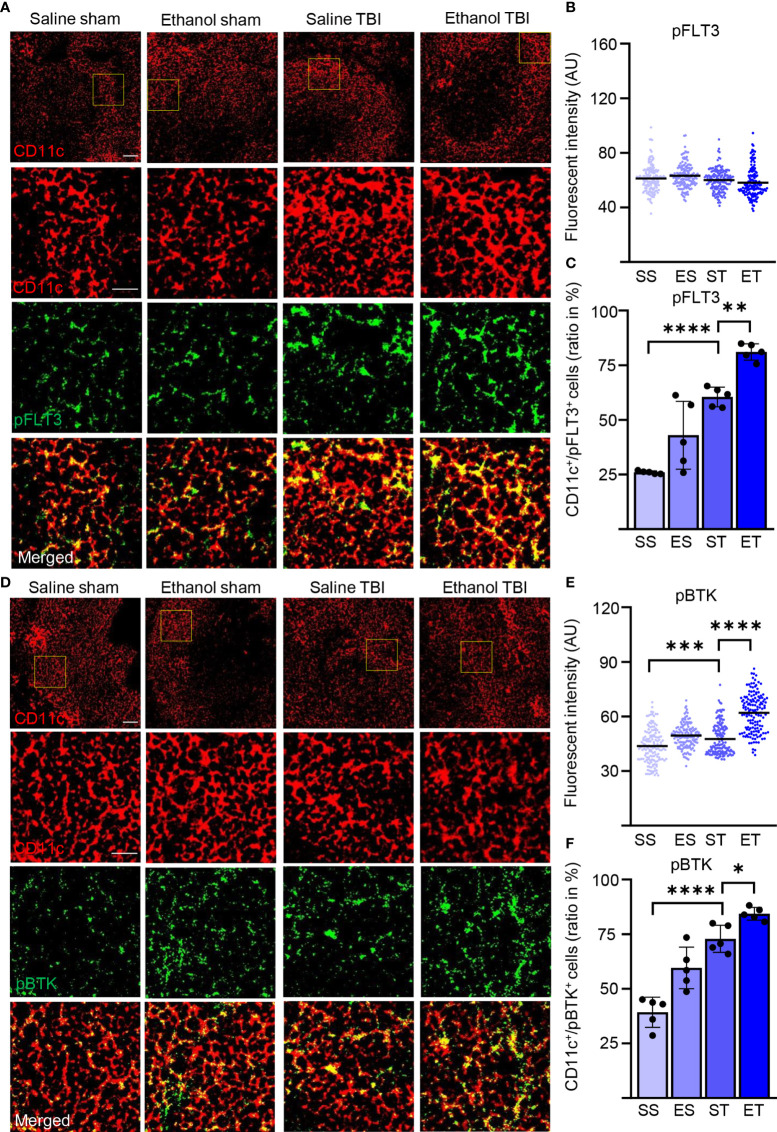
TBI induced the phosphorylation of FLT3 and the downstream signaling partner BTK is further enhanced by EI. Immunofluorescence staining of pFLT3 and pBTK with DC marker CD11c on thin spleen sections of saline sham (SS), ethanol sham (ES), saline TBI (ST), and ethanol TBI (ET)-treated mice 3 h after trauma. **(A–C)** Immunofluorescence staining of pFLT3 colocalized with CD11c resulted in no significant difference in fluorescence intensity between treatment groups (*p* = 0.06). However, the amount of FLT3^high^/CD11c+ cells revealed a significant increase after TBI (SS vs. ST; *p* < 0.0001) and a further significant enhancement in the ET group (ST vs. ET; *p* = 0.006). **(D–F)** Immunofluorescence staining of pBTK colocalized with CD11c resulted in a significant increase in fluorescence intensity upon TBI (SS vs. ST; *p* = 0.0008), with a further significant increase in the ET group (ST vs. ET; *p* = 0.006). Likewise, the number of BTK^high^/CD11c+ cells revealed a significant increase after TBI (SS vs. ST; *p* < 0.0001) with a further significant increase after ET (ST vs. ET; *p* < 0.0001). Data shown as scatter plots or bar plots with individual data points. Group size: SS *N* = 5, ES *N* = 5, ST *N* = 5, ET *N* = 5. *p < 0.05; **p < 0.01; ***p < 0.001; ****p < 0.0001. Scale bar overview: 50 µm; scale bar insert: 20 µm.

Our expression screening indicated a possible upregulation of FLT3 signaling in association with a cytokine pattern compatible with the involvement of DCs. Considering the well-known role of FLT3/FLT3L in regulating DC immunobiology (67), we sought direct confirmation of enhanced Flt3 engagement in splenic DCs upon TBI and ET. For this aim, we immunostained thin sections of the spleen from the four treatment groups for the pan-DC marker CD11c (21), for the pan-leukocyte marker CD45 (68), and phosphorylated FLT3 (pFLT3, Y589/591; **Figure 3A**). In agreement with the expression of CD45 in DC subpopulations (69), >95% of CD11c+ cells were also CD45+ (**Supplementary Figures 1D, E** **)**. Phospho-FLT3 immunoreactivity was highly inhomogeneous in the spleen sections, with a comparatively small number of cells highly positive for pFLT3 localized around follicles and a minor number of cells with moderate pFLT3 immunoreactivity scattered in the parenchyma. Initial immunostainings revealed that CD11c density was not altered by TBI or concomitant EI (two-way ANOVA, *F*_(3, 12)_ = 0.9360; *p* = 0.45; **Supplementary Figures 1B, C** **)**. Co-immunostaining with CD11c and CD45 revealed that nearly all the pFLT3^high^ cells (upon binning) were CD45+ (**Supplementary Figures 1F, G** **)**. However, when investigating the density of pFLT3^high^ cells in CD45+/CD11c− subpopulations (labeling leukocytes other than DC), we found no significant difference among treatment groups (two-way ANOVA, *F*_(3, 12)_ = 0.8446; *p* = 0.50; **Supplementary Figures 1F, G** **)**. Although the fluorescence intensity of pFLT3 in CD11c+ DCs was not altered across the four treatment groups (two-way ANOVA, *F*_(3, 588)_ = 2.541; *p* = 0.06; **Figure 3B**), the number of pFLT3^high^/CD11c+ cells was significantly affected by TBI and treatment (two-way ANOVA, *F*_(3, 12)_ = 45.84; *p* < 0.0001), and *post-hoc* comparison (Tukey corrected) revealed a significant increase in pFLT3^high^/CD11c+ cells after TBI alone (SS vs. ST; 20% ± 1% vs. 48% ± 4%; *p* < 0.0001; **Figure 3C**) and, interestingly, a substantial further enhancement in ET (ST vs. ET; 48 ± 4% vs. 65 ± 3%; *p* = 0.0061; **Figure 3C**), confirming that ST alone and, in particular, ET strongly induce FLT3 signaling in DCs. In order to verify that the phosphorylation of the FLT3 receptor corresponded to the functional engagement of signal transduction pathways, we monitored the phosphorylation of Bruton’s tyrosine kinase (BTK), an established downstream target of FLT3 (70). Immunostaining of thin spleen sections for phosphorylated BTK (pBTK, Y223; **Figure 3D**) revealed a pattern closely resembling that of pFLT3. The fluorescence intensity of pBTK in CD11c+ cells showed a significant difference between treatment groups (two-way ANOVA, *F*_(3, 444)_ = 123.5; *p* < 0.0001), and a *post-hoc* comparison (Tukey corrected) showed a significant increase after TBI (SS vs. ST; 44 ± 9 vs. 48 ± 9; *p* = 0.0008; **Figure 3E**). ET resulted in a further increase of pBTK fluorescence intensity (ST vs. ET; 48 ± 9 vs. 62 ± 11; *p* < 0.0001; **Figure 3E**). Likewise, quantification of the number of pBTK^high^/CD11c+ cells revealed a significant effect in the treatment groups (two-way ANOVA, *F*_(3, 12)_ = 68.07; *p* < 0.0001), due to the massive increase of pBTK^high^/CD11c+ cells in the ST compared with the SS group (Tukey’s *post-hoc*; SS vs. ST; 31% ± 6% vs. 58% ± 5%; *p* < 0.0001; **Figure 3F**), which was further increased in the ET group (ST vs. ET; 58% ± 5% vs. 67% ± 2%; *p* = 0.022; **Figure 3F**). Taken together, these data demonstrate that TBI causes fast (3 h) elevation of the phosphorylation of the FLT3 receptor (and its downstream target BTK) in splenic DCs and that this effect is substantially amplified by concomitant EI. Since CD11c+ cells were nearly always CD45+, we performed further investigations using the CD11c marker alone to identify DC.

### TBI and Concomitant EI–TBI Strongly Induce Protein Synthesis in Splenic DCs

**Figure 4:**
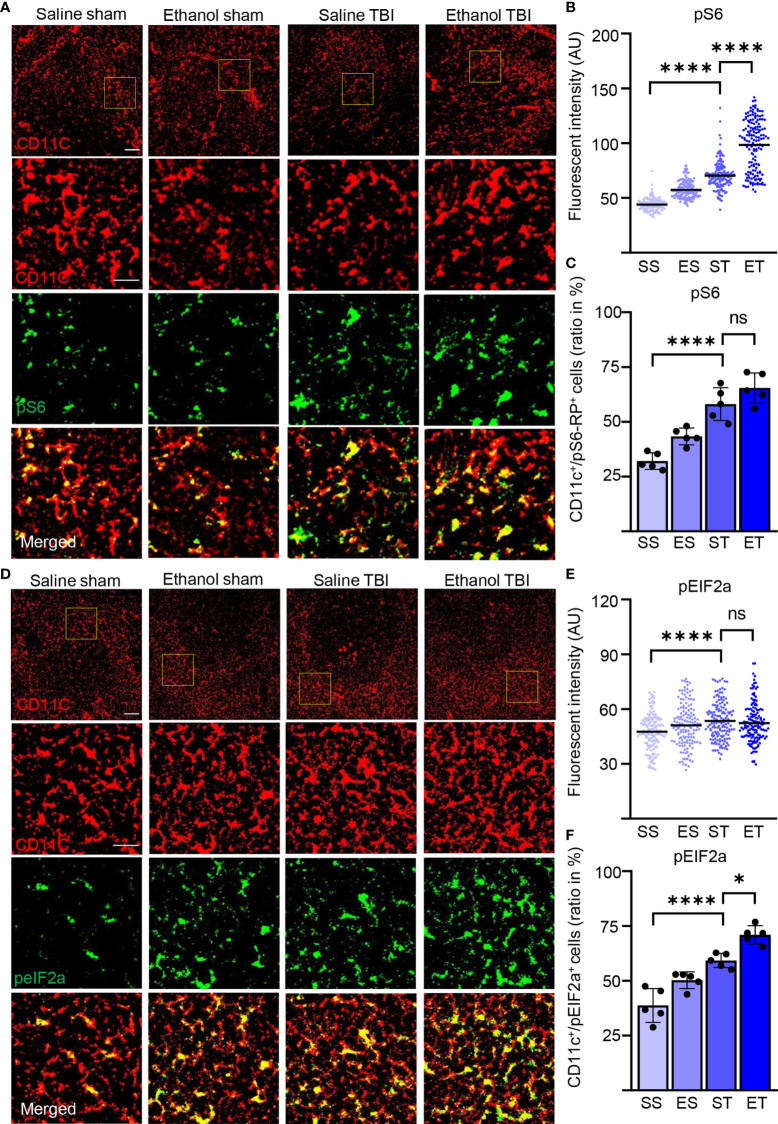
TBI induced the metabolic rate and protein synthesis is further enhanced by EI. Immunofluorescence staining of pS6-RP and peIF2A with DC marker CD11c on thin spleen sections of saline sham (SS), ethanol sham (ES), saline TBI (ST), and ethanol TBI (ET)-treated mice 3 h after trauma. **(A–C)** Immunofluorescence staining of pS6-RP colocalized with CD11c resulted in a significant increase in fluorescence intensity upon TBI (SS vs. ST; *p* < 0.0001), with a significant enhancement in the ET group (ST vs. ET; *p* < 0.0001). The amount of pS6-RP^high^/CD11c+ cells revealed a significant increase after TBI (SS vs. ST; *p* < 0.0001), but no significant difference in the ET group (ST vs. ET; *p* = 0.34). **(D–F)** Immunofluorescence staining of peIF2A colocalized with CD11c resulted in a significant increase in fluorescence intensity upon TBI (SS vs. ST; *p* < 0.0001), with no difference in the ET group (ST vs. ET; *p* = 0.78). The number of peIF2A^high^/CD11c+ cells revealed a significant increase after TBI (SS vs. ST; *p* = 0.0001), with a further significant increase in the ET group (ST vs. ET; *p* = 0.0107). Data shown as scatter plots or bar plots with individual data points. Group size: SS *N* = 5, ES *N* = 5, ST *N* = 5, ET *N* = 5. *p < 0.05; ****p < 0.0001; ns, not significant. Scale bar overview: 50 µm; scale bar insert: 20 µm.

Activation of DCs is characterized by a substantial remodeling of their metabolic rates and is particularly associated with the upregulation of protein synthesis (71) which, in turn, brings about the long-term adaptation of the cellular metabolism to the immune function (72). Since FLT3 is a strong regulator of mTor (73), a major driver of protein synthesis, we set out to determine if the upregulation of FLT3 signaling observed upon TBI, and enhanced by ET, was accompanied by the activation-associated increase in protein synthesis. First, we considered the levels of phosphorylated S6 ribosomal protein (pS6), a proxy of mTOR activation directly involved in increasing ribosomal translation of mRNA. Immunostaining of thin spleen sections for phosphorylated-S6 (pS6, S235/236; **Figure 4A**) revealed a diffuse immunopositivity in the cytoplasm in nearly every cell; however, upon TBI, CD11c+ cells stood out for having a massive increase in pS6 immunofluorescence intensity (two-way ANOVA, *F*_(3, 465)_ = 418.3; *p* < 0.0001). The *post-hoc* comparison (Tukey corrected) showed a significant increase after TBI (SS vs. ST; 44 ± 6 vs. 70 ± 13; *p* < 0.0001; **Figure 4B**) and further enhanced in the ET group (ST vs. ET; 70 ± 13 vs. 98 ± 23; *p* < 0.0001; **Figure 4B**). This effect corresponded to the significant increase in the number of CD11c+ displaying high levels of pS6 (two-way ANOVA, *F*_(3, 12)_ = 26.09; *p* < 0.0001) due to the substantial elevation of pS6 occurring after TBI (Tukey’s *post-hoc*; SS vs. ST; 32% ± 4% vs. 58% ± 8%; *p* = 0.0002; **Figure 4C**) but not further increased in ET compared with ST alone (ST vs. ET; 58% ± 8% vs. 65% ± 7%; *p* = 0.34; **Figure 4C**). Thus, ET magnifies the increased pS6 levels in all sensitive CD11c+ cells upon TBI but does not increase the number of cells responding to TBI itself.

Furthermore, the levels of the cellular stress-related phospho-eIF2A (71, 74) show a significant difference in fluorescence intensity in CD11c+ cells within treatment groups (two-way ANOVA, *F*_(3, 444)_ = 8.553; *p* < 0.0001, **Figure 4D**) due to a significant increase upon TBI (Tukey’s *post-hoc*; SS vs. ST; 48 ± 10 vs. 54 ± 10; *p* < 0.0001; **Figure 4E**), again with no difference between ET and ST (ST vs. ET; 54 ± 10 vs. 52 ± 11; *p* = 0.78; **Figure 4E**). Colocalization of peIF2A^high^ cells with CD11c+ cells reveals a significant difference within treatment groups (two-way ANOVA, *F*_(3, 12)_ = 40.50; *p* < 0.0001). The *post-hoc* comparison (Tukey corrected) shows a significant increase after TBI (SS vs. ST; 39% ± 8% vs. 60% ± 4%; *p* = 0.0001; **Figure 4F**) with a further increase in ET compared with ST alone (ST vs. ET; 60% ± 4% vs. 67% ± 6%; *p* = 0.01; **Figure 4F**). Thus, TBI induces a substantial increase in protein synthesis, with some alteration of cap-dependent translation in DCs, and this effect is amplified by EI.

### TBI and EI Cooperate to Enhance the Maturation of Splenic DCs

**Figure 5:**
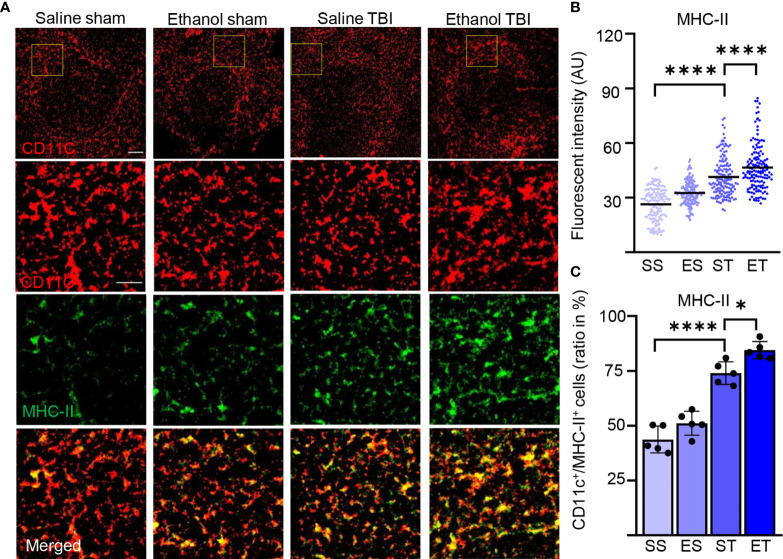
TBI induces antigen presentation on DCs, which is further enhanced by EI. Immunofluorescence staining of MHC-II with DC marker CD11c on thin spleen sections of saline sham (SS), ethanol sham (ES), saline TBI (ST), and ethanol TBI (ET)-treated mice 3 h after trauma. **(A–C)** Immunofluorescence staining of MHC-II colocalized with CD11c resulted in a significant increase in fluorescence intensity upon TBI (SS vs. ST; *p* < 0.0001), with a significant enhancement in the ET group (ST vs. ET; *p* < 0.0001). Likewise, the amount of MHC-II^high^/CD11c+ cells revealed a significant increase after TBI (SS vs. ST; *p* < 0.0001), with a significant enhancement in the ET group (ST vs. ET; *p* = 0.045). Data shown as scatter plots or bar plots with individual data points. Group size: SS *N* = 5, ES *N* = 5, ST *N* = 5, ET *N* = 5. *p < 0.05; ****p < 0.0001. Scale bar overview: 50 µm; scale bar insert: 20 µm.

DC maturation occurs on stimulation with pathogen-associated molecular patterns or danger-associated molecular patterns (PAMPs or DAMPs) (75, 76). Maturation of DCs is accompanied by an increase of antigen presentation and by lysosomal activity. Antigen presentation of DCs (and macrophages) can be visualized by quantifying MHC-II expression in CD11c+ cells (77). Furthermore, enhanced antigen presentation in DC has been associated with increased phagocytic and lysosomal activity, and upregulation of lysosomal markers, including CD68 and LAMP1, has been associated with increased antigen presentation and induction of T-cell responses (78–81). We wondered if the signaling events and metabolic reprogramming observed in splenic DCs after TBI also corresponded to the appearance of a “mature” APC phenotype. Spleen sections were stained for MHC-II and CD11c (**Figure 5A**), which reveals a high amount of MHC-II in DCs upon TBI, resulting in a significant increase of fluorescence intensity within treatment groups (two-way ANOVA, *F*_(3, 444)_ = 118.0; *p* < 0.0001). *Post-hoc* analysis (Tukey corrected) indicated a significant increase after TBI (SS vs. ST; 26 ± 9 vs. 41 ± 11; *p* < 0.0001; **Figure 5B**) with a further increase in the ET group (ST vs. ET; 41 ± 11 vs. 45 ± 13; *p* < 0.0001; **Figure 5B**). Furthermore, when assessing the density of colocalizing MHC-II^high^ with CD11c+ cells, we found a significant increase within treatment groups (two-way ANOVA, *F*_(3, 12)_ = 60.83; *p* < 0.0001) due to a massive increase upon TBI (Tukey corrected; SS vs. ST; 44% ± 6% vs. 74% ± 5%; *p* < 0.0001; **Figure 5C**) and a further increase in the ET group (ST vs. ET; 74% ± 5% vs. 84% ± 4%; *p* = 0.045; **Figure 5C**). Thus, both the number of CD11c+ cells expressing MHC-II and the levels of such expression increase upon TBI and are further increased by EI.

**Figure 6:**
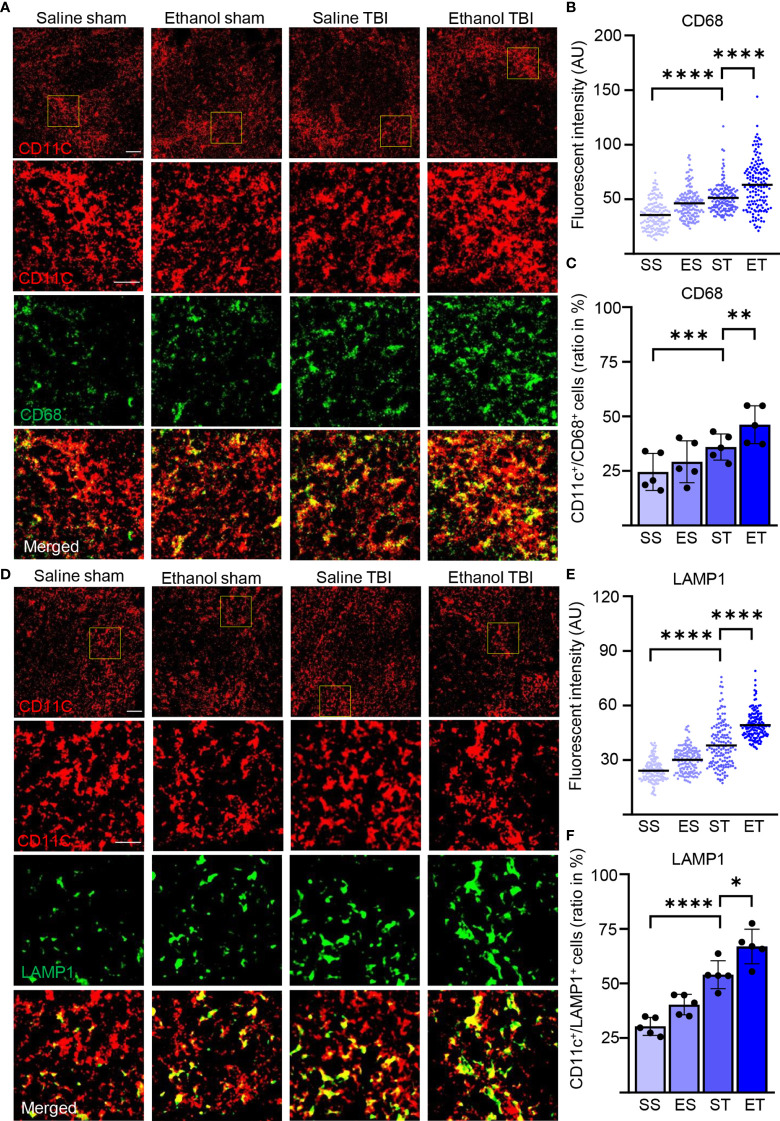
Lysosomal activity in DCs is increased by TBI and further enhanced by EI. Immunofluorescence staining of CD68 and LAMP1 with DC marker CD11c on thin spleen sections of saline sham (SS), ethanol sham (ES), saline TBI (ST), and ethanol TBI (ET)-treated mice 3 h after trauma. **(A–C)** Immunofluorescence staining of CD68 colocalized with CD11c resulted in a significant increase in fluorescence intensity upon TBI (SS vs. ST; *p* < 0.0001), with a significant enhancement in the ET group (ST vs. ET; *p* < 0.0001). The amount of CD68^high^/CD11c+ cells revealed a significant increase after TBI (SS vs. ST; *p* = 0.0007), with a significant enhancement in the ET group (ST vs. ET; *p* = 0.002). **(D–F)** Immunofluorescence staining of LAMP1 colocalized with CD11c resulted in a significant increase in fluorescence intensity upon TBI (SS vs. ST; *p* < 0.0001), with a significant increase in the ET group (ST vs. ET; *p* < 0.0001). The number of LAMP1^high^/CD11c+ cells revealed a significant increase after TBI (SS vs. ST; *p* = 0.0007), with a further significant increase in the ET group (ST vs. ET; *p* = 0.01). Data shown as scatter plots or bar plots with individual data points. Group size: SS *N* = 5, ES *N* = 5, ST *N* = 5, ET *N* = 5. *p < 0.05; **p < 0.01; ***p < 0.001; ****p < 0.0001. Scale bar overview: 50 µm; scale bar insert: 20 µm.

We also assessed the lysosomal activity of splenic DCs by investigating CD68 and LAMP1 in our samples (82, 83). Immunostaining of CD68 and CD11c (**Figure 6A**) on spleen sections revealed a significant difference in intensity within treatment groups (two-way ANOVA, *F*_(3, 444)_ = 79.68; *p* < 0.0001) with a strong increase after TBI (Tukey corrected, SS vs. ST; 36 ± 13 vs. 51 ± 13; *p* < 0.0001; **Figure 6B**) and an even further enhancement in the ET group (ST vs. ET; 51 ± 13 vs. 63 ± 22; *p* < 0.0001; **Figure 6B**). When assessing the density of CD68^high^ cells colocalized with CD11c+ DCs, the treatment resulted in a significant difference within groups (two-way ANOVA, *F*_(3, 12)_ = 41.25; *p* < 0.0001) due to TBI which resulted in an increase in the amount of CD68^high^/CD11c+ cells in the spleen (SS vs. ST; 25% ± 9% vs. 36% ± 6%; *p* = 0.0007; **Figure 6C**) and a further significant increase in ET (ST vs. ET; 36% ± 6% vs. 46% ± 9%; *p* = 0.002; **Figure 6C**). Similarly, immunostaining of LAMP1 and CD11c (**Figure 6D**) on spleen sections revealed a significant effect on intensity within treatment groups (two-way ANOVA, *F*_(3, 444)_ = 214.9; *p* < 0.0001) due to a significant increase upon TBI (Tukey corrected; SS vs. ST; 24 ± 6 vs. 38 ± 12; *p* < 0.0001; **Figure 6E**), and a further significant increase was detected in the ET group (ST vs. ET; 38 ± 12.3 vs. 49.2 ± 7.6; *p* < 0.0001; **Figure 6E**). The density of LAMP1^high^ cells colocalized with CD11c+ cells exhibited a significant effect within the treatment groups (two-way ANOVA, *F*_(3, 12)_ = 43.02; *p* < 0.0001). The *post-hoc* comparison within the treatment groups (Tukey corrected) showed a strong significant upregulation after TBI (SS vs. ST; 30% ± 4% vs. 54% ± 6%; *p* = 0.0007; **Figure 6F**) and a further significant enhancement in ET (ST vs. ET; 54% ± 6% vs. 67% ± 8%; *p* = 0.01; **Figure 6F**). The convergence of MHC-II, LAMP1, and CD68 upregulation indicates that upon TBI, APC functions are upregulated in splenic DCs and they are further enhanced by concomitant EI.

### TBI Strongly Induces the Immunogenic Function of Splenic DCs

**Figure 7:**
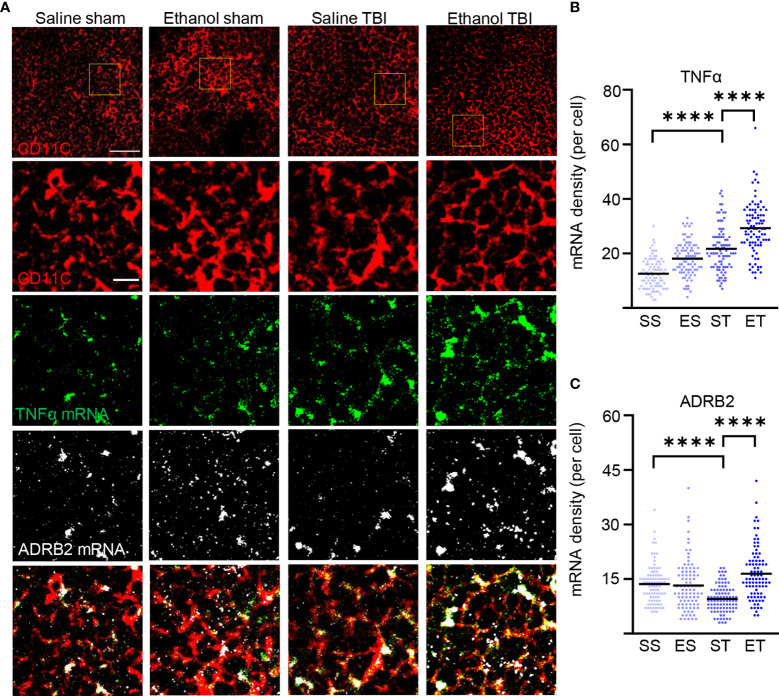
TBI enhances TNF-α expression in splenic DCs and EI shows high sensitivity to adrenergic dampening. Fluorescence *in-situ* mRNA hybridization of TNF-α and beta-2 adrenergic receptor (ADRB2) with co-staining of DC marker CD11c on thin spleen sections of saline sham (SS), ethanol sham (ES), saline TBI (ST), and ethanol TBI (ET)-treated mice 3 h after trauma. **(A, B)** Fluorescence *in-situ* hybridization of TNF-α resulted in a significant increase in mRNA density upon TBI (SS vs. ST; *p* < 0.0001), with a significant enhancement in the ET group (ST vs. ET; *p* < 0.0001). **(A–C)** Fluorescence *in-situ* hybridization of ADRB2 resulted in a significant decrease in mRNA density upon TBI (SS vs. ST; *p* < 0.0001); however, ET shows a significant increase (ST vs. ET; *p* < 0.0001). Data shown as scatterplots. Group size: SS *N* = 5, ES *N* = 5, ST *N* = 5, ET *N* = 5. ****p < 0.0001. Scale bar overview: 50 µm; scale bar insert: 10 µm.

The increased antigen presentation and lysosomal activity on CD11c+ DC, shown by the increase in MHC-II, CD68, and LAMP1, strongly suggest that TBI and EI induce a rapid maturation of splenic DC. However, the functional aspects of these mature DCs after TBI remain unclear. Initial evidence suggested that immature DCs are tolerogenic to T cells and mature DCs increase T-cell response and immunity (84, 85); however, other evidence points toward mature DCs with tolerogenic function (86, 87). To further explore the immunostimulatory phenotype of splenic DC in trauma, we assessed the expression levels of TNF-α in CD11c+ DCs. TNF-α is upregulated in DC by interaction with antigens and by stimulation of TLRs, and it is a major inducer of T-cell responses (88–90). We also considered the expression of the beta-2 adrenergic receptor, a marker associated with tolerogenic DC (91–93), under the hypothesis that expression of beta-2 adrenergic receptor and TNF-α should be inversely correlated. Fluorescence single mRNA *in-situ* hybridization was performed on spleen sections for ADRB2 and TNF-α (**Figure 7A**). Density analysis of single mRNA TNF-α in CD11c+ DCs revealed a significant difference within treatment groups (two-way ANOVA, *F*_(3, 267)_ = 79.5; *p* < 0.0001), due to a strong increase after TBI (Tukey corrected, SS vs. ST; 13 ± 6 vs. 22 ± 8; *p* < 0.0001; **Figure 7B**), with a further increase in the ET group (ST vs. ET; 22 ± 8 vs. 29 ± 10; *p* < 0.0001; **Figure 7B**). Furthermore, when assessing ADRB2 in CD11c+ DCs, density analysis revealed a significant difference within treatment groups (two-way ANOVA, *F*_(3, 267)_ = 18.99; *p* < 0.0001), due to a significant decrease after TBI (Tukey corrected, SS vs. ST; 14 ± 5 vs. 10 ± 4; *p* < 0.0001; **Figure 7C**). Interestingly, the ET group showed a strong increase in ADRB2 compared with the ST group (ST vs. ET; 10 ± 4 vs. 16 ± 7; *p* < 0.0001; **Figure 7C**). These data suggest that TBI induces an increase in immunogenic function of splenic DCs, shown by the increased TNF-α expression and decreased ADRB2 expression. Upon EI, the immunogenic function is further enhanced, shown by the increased TNF-α expression; however, the increase of ADRB2 suggests a simultaneous high sensitivity to adrenergic dampening of inflammation after EI.

## Discussion

Our data show that shortly (3 h) after TBI, splenic DCs undergo a maturation process that involves FLT3/FLT3L signaling, enhanced protein synthesis, increased phagocytotic and lysosome activity as well as upregulated expression of MHC-II, and finally, increased inflammatory properties, shown by TNF-α expression. Most notably, in the case of concomitant high-dose EI, the maturation process is enhanced, with increased expression of FLT3L and larger fractions of CD11c+ cells displaying elevated protein synthesis and signs of immune function activation, however with a simultaneous increased ADRB2 expression. Thus, not only does the TBI set in motion events that influence an important compartment of the systemic immune response, and even though concomitant EI is capable of substantially amplifying these cascades, it also simultaneously shows a rapid autonomic innervation.

Maturation of DCs is conceptualized as the phenotypic change from a state characterized by high endocytic capacity, low expression of co-stimulatory molecules and MHC-II, and weak induction of T-cell responses (immature dendritic cells) to a state of downregulated phagocytosis, high expression of MHC-I and MHC-II, and effective stimulation of naive T cells (77). Furthermore, maturation of DCs also involves a substantial remodeling of their metabolism, with increased mTOR-dependent protein synthesis (71, 73) and increased use of glycolytic pathways (94). DC maturation is also associated with a significant modification in the protein degradation flux, with upregulation of lysosomal markers such as LAMP3 (78) but reduced autophagy (95). Furthermore, the maturation process is intertwined with the upregulation of cytokine secretion such as TNF-α (88) and IL-12 (96). Therefore, with the demonstration of increased FLT3 phosphorylation, upregulation of protein synthesis markers, and increased expression of MHC-II and lysosomal proteins LAMP1 and CD68, we believe to provide substantial evidence to state that TBI induces a quick upregulation of the splenic DC maturation process, from steady-state cells to effective APCs. Several other markers are commonly applied to the study of DC maturation, such as CD40, CD80, and CD86 (97), which are often upregulated along with MHC-II [e.g., (98–100)]. These markers were not included in the present study and further characterization of the phenotype of splenic DC cells activated upon TBI may reveal their dynamics.

What drives such an induction of maturation? The maturation process of DCs is set in motion, among others, by PAMPs or DAMPs (75, 76), i.e., proteins released either by bacterial or viral pathogens, or by damaged tissue of the body. Indeed, the levels of brain tissue proteins and damaged markers are already elevated at 3 h in the serum of mice subject to TBI (including GFAP, NSE, S100B, and NFL) (37, 101). In particular, HMGB1, an alarmin located in the nucleus of neurons and glial cells and released upon brain tissue disruption (102, 103), is highly and rapidly elevated in serum after TBI (104, 105). Not only has HMGB1 been found to contribute to local neuroinflammation upon neurotrauma (106, 107), but also it is induced in non-cerebral tissues post-TBI and contributes to the subsequent systemic inflammation following TBI (10). Notably, HMGB1 is also a major inducer of DC maturation, an effect that appears to be relevant in the context of lung injury (through mTOR signaling) (108) and in liver injury (109). Nevertheless, recent evidence has demonstrated the strong involvement of the autonomic system in controlling splenic responses through adrenergic and cholinergic inputs (20, 22, 45, 110, 111). Moreover, the adrenergic activation of DCs is rather associated with limited expression of MHC-II and CD86 but strongly increases the secretion of IL-10 (112). Likewise, adrenergic stimulation of DCs substantially decreases the release of IL-12 and, in turn, suppresses the secretion of IFN-γ by Th1 lymphocytes (92). Thus, a role for the autonomic innervation in contributing to TBI-induced splenic DC maturation may take place along with the effect of systemic blood-borne cytokines.

What are the possible consequences of TBI-induced maturation of splenic DCs on local and systemic immune activation following brain injury? The net effect of the activation of the brain–spleen axis in TBI (either by circulating cytokines or by the dys-autonomia associated with TBI) seems to be detrimental, since immediate splenectomy results in an improved survival, reduced brain, and systemic cytokine response, ameliorated brain edema, and preservation of cognitive abilities in experimental rat TBI (4, 113). These effects were correlated with the decrease in NF-kB activation at the injury site (114). Similar beneficial effects of splenectomy have been reported in the context of spinal cord injury (SCI) (115) and stroke (20). However, the actual contribution of DC maturation may not necessarily be detrimental and may strongly depend on their activation status (immature, semi-mature, or mature) (77). It was shown that DCs pulsed with myelin-basic protein could drive a protective T-cell response in SCI (116); on the other hand, autoimmune responses following TBI are associated with detrimental outcomes (117). Interestingly, DC maturation is impaired in SCI patients (118). Thus, our findings suggest that TBI causes a rapid recruitment of DCs in the spleen. Given the central role of these cells as APCs, they may be substantial contributors to the systemic imbalance of immune functions following TBI.

What is the contribution of EI-driven enhanced DC maturation upon TBI? EI has been reported to be dose-dependently associated with a reduced inflammatory response (with decreased cellular inflammation and altered cytokine pattern) at the injury site (25–27) in murine TBI models. Importantly, EI also rapidly (already at 3 h) dampens the systemic inflammatory response triggered by TBI. In particular, in murine TBI, the levels of HMGB1 and IL-6 are decreased in the liver of mice subject to ET compared with TBI alone (but IL-1β is upregulated) (10). In contrast, in the lungs, EI decreases the levels of HMGB1, IL-6, IL-1β, and TNF-α, while it moderately increases IL-10 (10). In line with this evidence, EI is associated with reduced systemic IL-6 levels and less pronounced leukocytosis in human TBI patients (119) and in patients after major traumas (including TBI) (120), as well as increased levels of IL-10 (121). Our findings suggest that EI results in an increased number of splenic DCs undergoing a maturation process, driven by increased FLT3 phosphorylation and demonstrated by the larger fraction of CD11c+ cells displaying upregulated protein synthesis (pS6) and lysosomal markers (LAMP1, CD68) as well as the induction of high levels of TNF-α mRNA. It must be stressed that our model takes into consideration an acute consumption of a high dose of ethanol (“binge”), and therefore, our findings are not directly comparable with the reports of reduced DC ability to stimulate T cells upon chronic ethanol exposure (122, 123). Nevertheless, the combination of *in-vitro* and *in-vivo* data about the effects of EI on trauma-associated inflammation suggests an overall immunosuppressive effect of EI, in agreement with the impact of chronic ethanol on DC function. If this extrapolation is sound, then the enhanced maturation of splenic DCs seen in EI/TBI samples may result in a DC phenotype with inflammation-resolution properties. This hypothesis is supported by the observed upregulation of the beta-2 adrenergic receptor in DC. Nevertheless, the combination of increased TNF-α and ADRB2 mRNA (not previously described) may correspond to a peculiar state of activation characterized by high immune-stimulatory properties and, at the same time, quick dampening from autonomic innervation. Thus, the impact of EI-driven expansion of DC maturation may ultimately contribute to the reduced systemic immune reactivity seen in TBI upon ethanol intoxication.

The present work is not without limitations. First, the ultimate evaluation of the DC function would require an active immunization protocol *in vivo* or an *ex-vivo* naive T-cell stimulation assay, which was beyond the technical scope of the present project. Second, the use of CD11c+ marker does not distinguish the subpopulation of myeloid DC or the plasmacytoid DC; we have nevertheless maintained a consistent selection of the region of interest in correspondence of the marginal zone (124).

In conclusion, our findings show that induction of maturation markers in splenic DC takes place rapidly after TBI and is highly correlated with the phosphorylation of FLT3; we further demonstrate that concomitant EI amplifies the maturation process of splenic DC post-TBI. Thus, our findings identify DC as a new player in the immunomodulation occurring upon EI in TBI.

## Data Availability Statement

The raw data supporting the conclusions of this article will be made available by the authors, without undue reservation.

## Ethics Statement

The animal study was reviewed and approved by Regierungspräsidium Tübingen under licence number 1222.

## Author Contributions

FR and FoH conceived and designed the project. JZ, ZL, and SL performed the analysis of the spleen tissue. AC performed the TBI procedures. AL, TB, MH-L, FR, and FoH contributed to the analysis and the interpretation of the data. JZ, FR, MH-L, and FoH wrote the first draft of the manuscript. FR, FoH, JZ, ZL, SL, AL, TB, and MH-L contributed to the final version of the manuscript.

## Funding

This work was supported by the Deutsche Forschungsgemeinschaft as part of the Collaborative Research Center 1149 “Danger Response, Disturbance Factors and Regenerative Potential after Acute Trauma” (DFG No. 251293561). JZ was supported by the Chinese Scholarship Council (ID No. 202108080183). FR was also supported by the ERANET-NEURON initiative “External Insults to the Nervous System” as part of the MICRONET consortium (funded by BMBF: FKZ 01EW1705A).

## Conflict of Interest

The authors declare that the research was conducted in the absence of any commercial or financial relationships that could be construed as a potential conflict of interest.

## Publisher’s Note

All claims expressed in this article are solely those of the authors and do not necessarily represent those of their affiliated organizations, or those of the publisher, the editors and the reviewers. Any product that may be evaluated in this article, or claim that may be made by its manufacturer, is not guaranteed or endorsed by the publisher.

## Supplementary Material

Supplementary Figure 1 CD11c+ DC density is unaffected by TBI and EI. FLT3 phosphorylation remains unaltered in CD45+/CD11c- cells. Immunofluorescence staining of thin spleen sections of saline-sham (SS), ethanol-sham (ES), saline-TBI (ST) and ethanol-TBI (ET). **(A)** Immunofluorescence staining of CD11c in control groups reveals inhomogeneous distribution in thin spleen sections. With a high localisation around the follicles. **(B, C)** Density of CD11c+ cells remain unaltered 3h between treatment groups (p = 0.45). **(D, E)** Immunofluorescence staining of CD11c and CD45 shows that 95.3% of CD11c+ cells are CD45+. **(F, G)** immunofluorescence staining of CD45, CD11c and pFLT3 reveals unaltered phosphorylation levels of FLT3 in CD45+/CD11c- cells between treatment groups (p = 0.50). Data shown as barplots with individual data points. Group size: SS N = 5, ES N = 5, ST N = 5, ET N = 5. Scale bar overview A: 200 µm; scale bar insert A: 50 µm; scale bar overview B and D: 50 µm; scale bar insert D: 20 µm; scale bar overview B: 50 μm; scale bar insert F: 20 μm.

Supplementary Table 1 Primer sequences used for gene expression analysis (RT-qPCR).

Supplementary Table 2 Primary and secondary antibodies used for immunostaining (IF).

Supplementary Data File 1 Correlation matrices corresponding to **Figures 1**, **2**. Correlation matrices to investigate possible correlation between cytokine and chemokine expression patterns within the treatment groups saline-sham (SS), ethanol-sham (ES), saline-TBI (ST) and ethanol-TBI (ET). A significant correlation in both ST and ET was detected between *IL-19* and *IL-17* (ST: p < 0.0001; ET: p < 0.0001), *IL-19* and *IL-23a* (ST: p = 0.0011; ET: p < 0.0001), *IL-19* and *IFN-y* (ST: p < 0.0001; ET: p < 0.0001), *IL-17* and *IL-23a* (ST: p = 0.0002; ET: p < 0.0001), *IL-17* and *IFN-y* (ST: p < 0.0001; ET: p < 0.0004) and between *IL-23a* and *IFN-y* (ST: p = 0.0011; ET: p = 0.0011). *FLT3L* was significantly correlated with *FLT3* in the ET group, but not in the ST group (p = 0.0028). A significant correlation only in ET, but not ST, was detected between *CCL2* and *CCL1* (p = 0.012), *CCL2* and *CCL20* (p = 0.018), CCL*3* and *CCL1* (p = 0.0005), *CCL3* and *CCL22* (p = 0.049), *CCL3* and *CCL20* (p = 0.015), *CCL4* and *CXCL10* (p = 0.007), *CCL4* and *CCL20* (p = 0.044), *CCL4* and *CCL24* (p = 0.005). Data shown as correlation matrices with Pearson r coefficient and p-values for every correlation individually.
