# Supplementary figures and images for: Fast Maturation of Splenic Dendritic Cells Upon TBI Is Associated With FLT3/FLT3L Signaling
Source: Front Immunol. 2022 Feb 24;13:824459. doi: 10.3389/fimmu.2022.824459 (PMC8907149; doi:10.3389/fimmu.2022.824459)

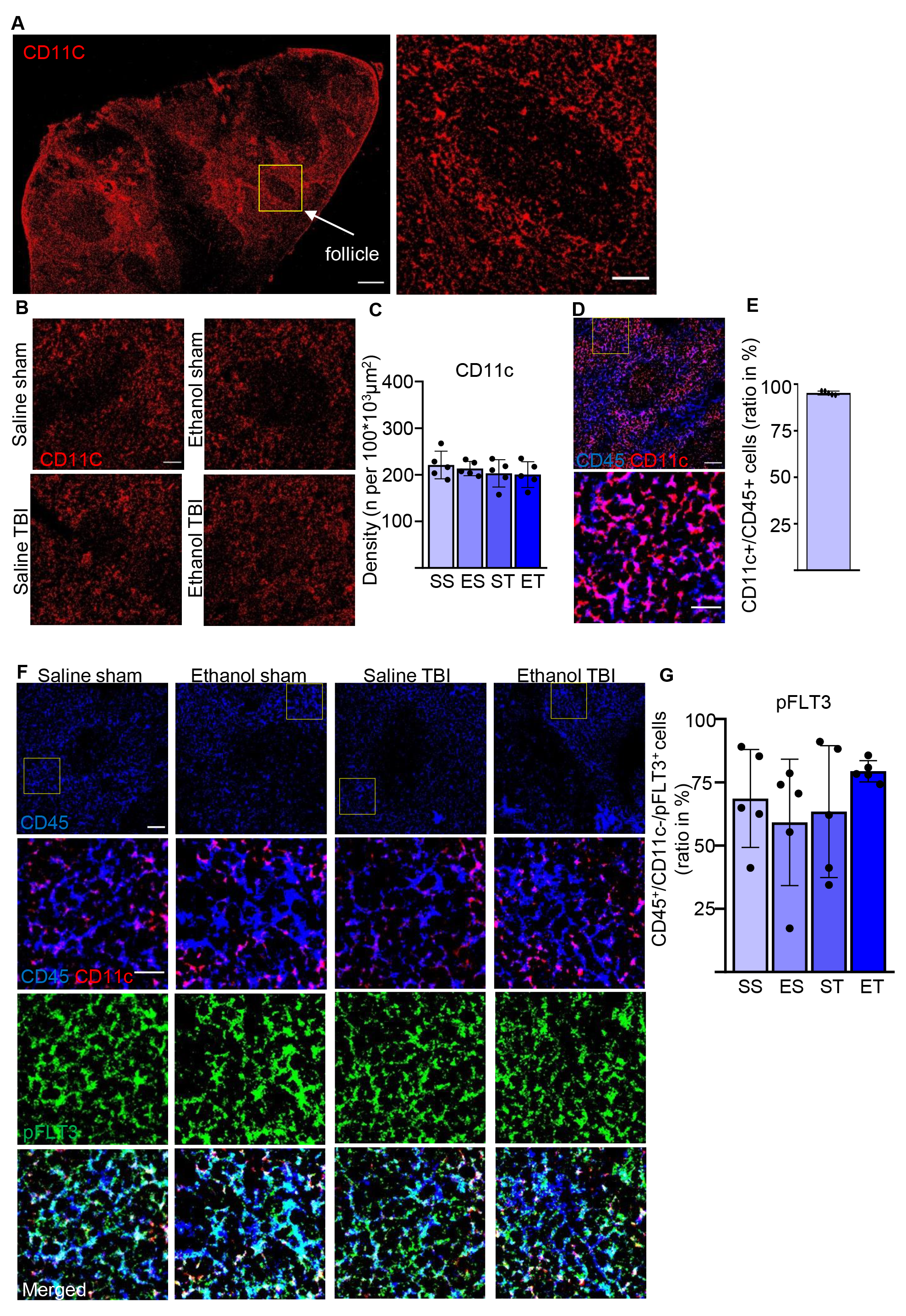

Supplement: Supplementary Figure 1 — CD11c+ DC density is unaffected by TBI and EI. FLT3 phosphorylation remains unaltered in CD45+/CD11c- cells. Immunofluorescence staining of thin spleen sections of saline-sham (SS), ethanol-sham (ES), saline-TBI (ST) and ethanol-TBI (ET). (A) Immunofluorescence staining of CD11c in control groups reveals inhomogeneous distribution in thin spleen sections. With a high localisation around the follicles. (B, C) Density of CD11c+ cells remain unaltered 3h between treatment groups (p = 0.45). (D, E) Immunofluorescence staining of CD11c and CD45 shows that 95.3% of CD11c+ cells are CD45+. (F, G) immunofluorescence staining of CD45, CD11c and pFLT3 reveals unaltered phosphorylation levels of FLT3 in CD45+/CD11c- cells between treatment groups (p = 0.50). Data shown as barplots with individual data points. Group size: SS N = 5, ES N = 5, ST N = 5, ET N = 5. Scale bar overview A: 200 µm; scale bar insert A: 50 µm; scale bar overview B and D: 50 µm; scale bar insert D: 20 µm; scale bar overview B: 50 μm; scale bar insert F: 20 μm. [file Image_1.tif]
